# *Arum palaestinum* delays hepatocellular carcinoma proliferation through the PI3K-AKT-mTOR signaling pathway and exhibits anticoagulant effects with antimicrobial properties

**DOI:** 10.3389/fphar.2023.1180262

**Published:** 2023-06-01

**Authors:** Majdi Dwikat, Johnny Amer, Nidal Jaradat, Ahmad Salhab, Ahmad Abdal Rahim, Mohammad Qadi, Aseel Aref, Mustafa Ghanim, Haya Murad, Ali Modallal, Kawkab Shalabni

**Affiliations:** ^1^ Department of Allied Sciences, Faculty of Medicine and Health Sciences, An-Najah National University, Nablus, Palestine; ^2^ Department of Pharmacy, Faculty of Medicine and Health Sciences, An-Najah National University, Nablus, Palestine; ^3^ Department of Biomedical Sciences, Faculty of Medicine and Health Sciences, An-Najah National University, Nablus, Palestine

**Keywords:** *Arum palaestinum*, antimicrobial, anticoagulant, anti-cancer, alpha-fetoprotein

## Abstract

**Background:** *Arum palaestinum* Boiss (AP) is a wild plant in Palestine whose leaves have a long history as food and medicine in Middle Eastern countries. The current study aimed to evaluate the biological characteristics of AP flower extract, including its antimicrobial and coagulation cascade activities and its effects on anticancer molecular pathways.

**Methods:** The antimicrobial activity of the aqueous extract of AP flowers was assessed using a microdilution assay against eight pathogens. The coagulation properties were assessed by prothrombin time (PT), activated partial thromboplastin time (aPTT), and thrombin time (TT) tests using standard hematological methods. The biological effects of AP on hepatocellular carcinoma were measured by assessing the impact of AP on cell cycle, proliferation (CFSE), apoptosis (annexin-v+/PI), and tumorigenicity (αFP and HBsAg), as well as its effects on the PI3K-AKT-mTOR molecular signaling pathway.

**Results:** The antimicrobial screening results revealed that the aqueous extract of AP had potent antibacterial effects against *P. vulgaris* and *E. faecium* compared to ampicillin, with MIC values of 6.25, 6.25, and 18 μg/mL, respectively. Moreover, the AP aqueous extract exerted anticoagulant activity, with significant prolonged results in the aPTT and TT tests (25 μg/mL and 50 μg/mL, respectively) and slightly prolonged results in the PT test (50 μg/mL). The anticancer results indicated a delay in the cell cycle through decreased cell proliferation rates following incubation with AP fractions. The effect of the aqueous fraction was most evident in a delay in the S phase. The aqueous and DMSO fractions maintained the cells in the G2-M phase, similar to the DOX, while the flower extract in methanol accelerated the cells in the G2-M phase, suggesting that AF flower extracts may have anti-cancer properties. The aqueous extract of AP 1) reduced secretions of HCC αFP by 1.55-fold and 3.3-fold at the 50 and 100 μg/mL concentrations, respectively (*p* = 0.0008); 2) decreased phosphorylation in the PI3K-AKT-mTOR signaling pathway (*p* < 0.05); and 3) shifted cells from necrosis to apoptosis by 50% and 70% at the 50 and 100 μg/mL concentrations, respectively (*p* < 0.05).

**Conclusion:** The results of this study showed the activities of the bioactive components for the treatment of infectious diseases and blood coagulation disorders, which could also be a potential therapeutic approach for delaying HCC tumorigenicity.

## Introduction

Herbal medicine is a traditional therapeutic method that many people have used in their communities to treat and prevent harmful diseases (Jaradat et al., 2017). This kind of treatment is based on the use of different plant parts such as leaves, flowers, seeds, bark, fruits, and roots to prepare medicinal remedies for disease control, prophylaxis, or cure, and later developing into synthetic or semisynthetic drugs based on the assumption that they are safer, more potent, selective, and have fewer side effects (Padayachee and Baijnath, 2020). Herbal medicine is thought to play an important role in drug discovery, mostly because it offers potential lead compounds, which result from several steps in the processing of plants, including isolation, purification, and molecular characterization (Anand et al., 2019).

Thus, one of the lead sources is plant secondary metabolites that are derived biosynthetically from plant primary metabolites. These secondary metabolites are bioactive chemicals that include alkaloids, glycosides, phenolic compounds, tannins, and terpenoids, which continue to be good model molecules in drug discovery due to their potential medicinal applications for the treatment of numerous conditions and diseases from migraines to cancers (Ahmed et al., 2017). Thus, herbal medicines help humanity to discover and develop medicines for many diseases, especially intractable ones (Lombardino and Lowe, 2004). The molecular process by which the body forms clots to avoid bleeding is the coagulation cascade. Platelets, endothelial cells, and leukocytes must be activated so that a suitable surface is provided for the adhesion of clotting proteins (Green, 2006). Blood coagulation cascade reactions are propagated by complex enzymes containing a vitamin K-dependent serine protease and an accessory cofactor protein, which are calcium-dependently assembled on the membrane surface (Butenas and Mann, 2002). The traditional view of the regulation of blood coagulation consists of extrinsic and intrinsic pathways. The extrinsic pathway depends on the initiation phase, which includes plasma factor VII/VIIa and transmembrane receptor tissue factor (TF), whereas the intrinsic pathway requires the amplification phase, which includes plasma FXI, FIX, and FVIII (Machman et al., 2007). Antibiotics are usually used to treat various sorts of microbial infections. Some microorganisms can develop more resistant strains by mutation or acquired resistance against some antibiotics. For example, most Gram-negative (−ve) are microorganisms inherently resistant to vancomycin. Bacteria are deemed resistant to an antibiotic if their development is not prevented by the maximum amount of the antibiotic that can be absorbed in the host dosage. However, microbial strains become resistant by the genetic alteration or/and the expression of proteins, usually by modification of the target site, reduction of drug accumulation, or by the inactivation of enzymes (Jamie, 2015). *Arum palaestinum* Boiss (AP) (Araceae) is a perennial herbaceous plant that is native to the Levant and other countries in the Mediterranean Basin. It grows up to 0.82 ft and blooms in the spring, between March and April. The plant is well recognized by its dark purplish-black spadix enclosed by a reddish-brown spathe (Afifi et al., 1999; Azab, 2017). In traditional Palestinian medicine, AP aqueous extract has been used for the treatment of bacterial infection, cough, cancer, constipation, intestinal worms, skin infections, blood circulation disorders, and renal stones (Azab, 2017). While isoorientin, vitexin, luteolin, apigenin, quercetin-3-O-beta-glucoside, quercetin, esculin, ferulic, and caffeic acids are the major recognized molecules in leaves of AP (Afifi et al., 1999), phytol, phytol acetate, linolenic, linoleic, and hexadecanoic acids, are the major compounds in the aerial parts of AP (Farid et al., 2015; Afifi et al., 2016). Few reports on the cytotoxic activities have used different selected fractions and pure compounds of AP to provide evidence for its strong antitumor activities on selected cell lines (Farid et al., 2015) and provided no indications of mechanistic approaches and molecular signaling pathways. To the best of the author’s knowledge, no previous studies have evaluated the antimicrobial and antiplatelet activities of AP, or its liver cancer tumorigenicity.

## Methods

### Collection of plant material

AP flowers were collected in April–May 2019 from the Tulkarem region in Palestine. The plant voucher specimens Pharm-PCT-246 were deposited in the Pharmacognosy Laboratory, Faculty of Pharmacy at An-Najah National University. The collected flowers were washed with distilled water and later completely dried in the shade at room temperature for 3 weeks. The dried parts were then ground into a fine powder using a mechanical blender and stored in tightly sealed special containers until use.

### Preparation of AP samples

The dried AP flowers were chopped into small pieces, and 100 g was boiled in 1 L of distilled water until the original volume was decreased to one-fourth. After that, the decoction was filtered (Macherey-Nagel, MN 617 and Whatman no. 1, United States), and the filtrate was placed into a freeze drier (Millrock Technology-BT85, China) apparatus until the aqueous extract turned into a solid powder. The dried extract was then kept in a well-closed container for further use. Next, the AP flower extracts were diluted in water, DMSO, and methanol solutions. To obtain the concentration of 100 μg/mL, 1 mg from each of the preserved extracts was taken. Then, it was diluted in 10 mL of the three solutions. Finally, 1 mL of the prepared dilution was obtained using a pipette.

### Blood sample collection and preparation

Citrated blood samples were collected from five healthy volunteers. None of the participants were taking any medications, especially anticoagulants, and they were non-smokers. One part of sodium citrate was mixed with nine parts of blood to obtain a ratio equal to 1:9. The citrated blood was centrifuged at 2,500 rpm for 15 min to obtain citrated-PPP (platelet-poor plasma). Prothrombin time (PT), activated partial thromboplastin time (aPTT), and thrombin time (TT) tests were conducted on the plasma within 2 h of blood collection. All results were obtained using a digital coagulation analyzer (coagulation analyzer Coa-DATA 4004, Germany). All measurements were conducted in duplicate, and 1% DMSO was used as a negative control (Omar et al., 2017).

### Prothrombin time (PT) test

The *in vitro* method was used for the test. In this test, 100 μL of pre-warmed plasma was incubated with 100 µL of plant extract for 5 min. All concentrations (50, 25, 5, and 1 μg/mL) of AP aqueous extract were tested. The clotting time was measured immediately after the addition of 200 µL of the pre-warmed thromboplastin reagent (Hemostat thromboplastin-SI. Human, Germany) (Omar et al., 2017).

### Activated partial thromboplastin time (aPTT) test

The *in vitro* method was used for the test. In this test, 100 µL of pre-warmed plasma was incubated with 100 µL of plant extract for 5 min. All concentrations (50, 25, 5, and 1 μg/mL) of AP aqueous extract were tested. After that, PTT reagent was added, and the mixture was incubated for 3 min. The clotting time was measured immediately after the addition of 50 µL of CaCl_2_ reagent (aPTT, Human, Germany) (Omar et al., 2017).

### Thrombin time (TT) test

The *in vitro* method was used for the test. In this test, 100 µL of pre-warmed plasma was incubated with 100 µL of plant extract for 5 min. All concentrations (50, 25, 5, and 1 μg/mL) of AP aqueous extract were tested. The clotting time was measured immediately after the addition of 100 µL of pre-warmed thrombin reagent (Hemostat Thrombin Time. Human, Germany) (Omar et al., 2017).

### Microbial isolates

The examined bacterial and fungal isolates were obtained from the American Type Culture Collection (ATCC), in addition to a clinically confirmed methicillin-resistant *Staphylococcus aureus* (MRSA) isolate. The selected species of microorganisms are frequently isolated in clinical settings in our region, and some possess multidrug resistance. The isolates included three Gram-positive strains, namely, *S. aureus* (ATCC 25923), MRSA, and *Enterococcus faecium* (ATCC 700221), and four Gram-negative strains, namely, *Klebsiella pneumoniae* (ATCC 13883), *Proteus vulgaris* (ATCC 700221), *Escherichia coli* (ATCC 25922), and *Pseudomonas aeruginosa* (ATCC 27853). The fungal isolates included *Candida albicans* (ATCC 90028).

### Antimicrobial test

The antimicrobial activities of the aqueous AP flower extract were assessed using the micro-broth dilution method. The aqueous extract was prepared using distilled water to a concentration of 100 μg/mL. The experiments were conducted in 96-well plates. The extract was subjected to 10, two-fold serial dilutions, from well 1 to well 10, in Mueller–Hinton broth for bacterial isolates and RPMI media for the fungal isolate to determine the minimum inhibitory concentrations (MICs) for all tested microorganisms. The bacterial suspensions were prepared by picking colonies from an overnight agar culture plate of the test organism, adding it to a test tube containing 5 mL of normal saline, adjusting the turbidity to 0.5 McFarland, and diluting in Mueller–Hinton broth (RPMI for *C. albicans*) to obtain a final bacterial concentration of 5 × 10^5^ cfu/mL (2.5 × 10^3^ cfu/mL for *C. albicans*) in each well. Well number 11 was used as a positive control containing bacteria and without AP extract. Well number 12 was used as a negative control containing media alone and without microbes and AP extract. The MIC was defined as the lowest concentration of an antimicrobial that inhibited the growth of a microorganism after an incubation period of 18–24 h (for plates inoculated with the test bacterial strains) and around 48 h (for plates inoculated with *C. albicans*)*.* Ciprofloxacin and ampicillin were used as reference antibacterial activity controls for our method, while fluconazole was used as a reference antifungal activity control (Balouiri et al., 2016).

### Cell culture

The Hep3B cell line was used to assess the biological impact of AP on cancer activity and alteration in cell molecular pathways. Hep3B has the same genotype, phenotype, and features of hepatocellular carcinoma (HCC). The normal healthy counterparts of primary hepatocytes were used as controls in our study. Hep3B cells are characterized by the secretion of α-fetoprotein (αFP) and HBsAg which are considered tumor markers. The αFP levels were assessed using a commercially available ELISA kit (R&D Systems, Inc., United States). The Hep3B cells and hepatocytes were cultured in RPMI-1640 medium enhanced with 1% penicillin, 1% streptomycin, 1% l-glutamine, and 10% fetal bovine serum adjusted to pH 7.2 using Dulbecco’s phosphate-buffered saline (DPBS). The cells were grown at 37°C in an ESCO cell culture incubator with a humidified atmosphere of 95% air and 5% CO_2_ at 37°C. The cells were treated with AP concentrations of 50 and 100 μg/mL for 48 h.

### Alpha-fetoprotein (αFP) detection

αFP is produced by regenerating liver cells. In chronic liver diseases such as hepatitis and cirrhosis, αFP levels may be chronically elevated. Very high concentrations of αFP may be produced by certain tumors. This characteristic makes the αFP test useful as a tumor marker. Increased αFP levels are found in many people with the most common type of liver cancer, hepatocellular carcinoma, and in a rare type of liver cancer that most commonly occurs in infants, hepatoblastoma. The secreted αFP concentrations in the Hep3B cell culture medium were detected using a Human alpha-Fetoprotein Quantikine ELISA Kit (R&D; DAFP00). Absorbance was measured at 450 nm on a Universal Microplate Reader.

### Cell proliferation assay

Hep3B and primary hepatocytes were seeded in 12-well plates (1 × 10^6^ cells/well) with RPMI containing 10% FBS. Each well was treated with and without AP at different concentrations for 48 h. After the cells were harvested, the cell numbers were counted using a hemocytometer, and CSFE MFI was identified using an LSR II-Fortessa reader.

### Primary hepatocyte isolation

The source of the primary human hepatocytes was from biopsies of human liver from patients with liver cancer obtained from normal histological tissue lobe. Briefly, the tissue was minced (<3 mm) on ice using two scalpels in a scissor motion. The tissue was then transferred to a specimen container containing pre-warmed EGTA buffer (HBSS, 0.5 mM EGTA, 0.5% fatty acid-free bovine serum albumin [BSA]) and agitated (100 rpm) in a water bath at 37°C with a shaking bed for 10 min. The tissue was then washed three times in HBSS to remove the remaining blood and EGTA. The tissue was then placed in pre-warmed digestion buffer (HBSS, 0.05% collagenase IV, 0.5% fatty acid-free BSA, 10 mM CaCl2) and agitated (100 rpm) in a water bath with a shaking bed for 30 min at 37°C. BSA was included in all digestion buffers to minimize cell damage and prevent the hemolysis of red blood cells (RBCs). The digested tissue was passed through a metal (tea) strainer, and the supernatant was collected and filtered through a 100-μm cell strainer. The supernatant was kept on ice. The remaining tissue was again digested in fresh digestion buffer. The cell suspensions were pooled and centrifuged (80 g for 5 min, 4°C), and the supernatant was discarded. The hepatocytes were washed twice in William’s E buffer. The cells were counted, and the viability was measured by trypan blue exclusion. The cells were then diluted to 1 million cells per ml in William’s E buffer containing supplements (1% non-essential amino acids [NEEA], 1% GlutaMAX™, 2% human serum, 100 nM dexamethasone, 100 nM insulin, and 0.375% fatty acid-free BSA). The hepatocytes were plated on type 1 collagen-coated plates at a density of 250,000/cm^2^. After cells had adhered (3–4 h), the media was removed and replaced with William’s E maintenance media (1% NEEA, 1% GlutaMAX™, 100 nM dexamethasone, 100 nM insulin, and 0.375% fatty acid-free BSA).

### Flow cytometry analysis

Following culture, the harvested Hep3B cells and primary hepatocytes were adjusted to 10^6^/mL in staining buffer (in saline consisting of 2% bovine albumin). For measurements of viability and apoptosis, fragmented DNA was stained using propidium iodide (PI), and phosphatidylserine was stained using annexin V-conjugated fluorescein isothiocyanate (FITC) according to the manufacturer’s instructions (Phosphatidyl Serine Detection Kit—FITC, IQP-116F). Apoptosis was marked as annexin-V (+) but propidium iodide (−). In contrast, viable cells were marked as annexin-V (−) but propidium iodide (−). Unstained controls were used in each of the experiments, such as IgG isotype and Fluorescence Minus One (FMO) controls. The analysis of the cell cycle by quantization of DNA content was achieved by propidium iodide staining. The cells were fixed in cold 70% ethanol at 4°C for at least 30 min. Next, the cells were washed 2X in phosphate-buffered saline (PBS) and centrifuged at 2000 rpm to dispose of the supernatant. To ensure that only DNA was stained, cells were also treated with ribonuclease (50 μL of 100 μg/mL RNase). The cells were stained with mouse anti-human HbsAg, rabbit anti-human pPI3K, rat anti-human pAKT, and mouse anti-human pmTOR (R&D). Data were analyzed on the flow cytometer (LSR II, Immuno-fluorometry systems, Mountain View, CA).

### Western blot analysis

Hep3B protein extracts were prepared in a homogenization buffer (50 mmol/L Tris–HCl [pH 7.6], 0.25% Triton-X 100, 0.15 M NaCl, 10 mM CaCl_2_, and complete mini EDTA-free protease inhibitor cocktail, Roche Diagnostics, Mannheim, Germany). Next, the proteins (30 μg per lane) were resolved on a 10% (w/v) SDS-polyacrylamide gel (Novex, Groningen, Netherlands) under reducing conditions. For immunoblotting, the proteins were transferred to a Protran membrane. The blots were then incubated for 1 h at room temperature in a blocking buffer containing 5% skim milk (w/v). Next, the blots were incubated overnight at 4°C with rabbit anti-human phosphorylated PI3K/AKT/mTOR (R&D System, Minneapolis, MN) diluted 1:1,000, and subsequently, with peroxidase-conjugated with anti-rabbit/mouse/rat (Abcam, Israel, diluted 1:5,000), for 1.5 h at room temperature. Immunoreactivity was detected using an ECL kit (Abcam, Israel).

### Immunofluorescence staining

For deparaffinization, paraffin-embedded sections were placed at 60°C for 15 min, incubated in xylene at room temperature for 15 min, and then transferred sequentially into 100% EtOH, 95% EtOH, 70% EtOH, and 50% EtOH for 4 min, respectively, at room temperature. The sections were rinsed in deionized water and stored in PBS. For antigen retrieval, we used a buffer (10 mM citrate, pH 6.2, 2 mM EDTA, and 0.05% Tween 20) for anti-albumin detection. Primary hepatocytes were outlined with 100 μL of KASBLOCK liquid blocker to minimize the volume of antibody solution needed for staining. The samples were then incubated overnight at 4°C with rabbit anti-human albumin (diluted 1:30) (IQ Products, Groningen, Netherlands). The samples were washed with PBS; next, secondary antibodies conjugated with Cy-3 were applied for 1 h at room temperature, and image capture was performed. DAPI was used for nucleus staining (blue). The samples were viewed and imaged with a Zeiss LSM 710 confocal laser-scanning system (Zeiss, Germany) attached to a Zeiss Axiovert 135M microscope, equipped with a Plan-Apochromat Zeiss 63X lens. An Alexa Fluor laser (552 nm) was used to detect red fluorescence.

### Statistical assessment

Statistical significance was determined by two-tailed unpaired Student’s t-test (for comparison between two groups) and chi-squared or one-way analysis of variance (one-way ANOVA with Newman–Keuls post-tests) for multiple groups with GraphPad Prism 5.0 (GraphPad Software, La Jolla, CA). *In vitro* experiments were repeated three times, with four sample replicates each. Data are represented as mean ± SEM.

## Results

### Antimicrobial activity

The antimicrobial activities of the crude AP aqueous extracts were determined using micro-dilution assays against selected microbial pathogens, including Gram-negative, Gram-positive, and fungal strains. The results showed that the aqueous AP extract has antibacterial activity compared with the positive antibacterial controls (ampicillin and ciprofloxacin) and no antifungal activity compared with the antifungal drug fluconazole as a positive control (Table 1). The aqueous AP extract exhibited an antibacterial effect against all the tested bacterial strains, with a strong antibacterial effect against *P. vulgaris* compared with ampicillin with MIC values of 6.25 and 18 μg/mL, respectively. AP also showed antibacterial activity against *E. faecium*, with MIC values of 6.25 μg/mL. However, the antibacterial MIC values for AP were lower than those for ciprofloxacin.

**TABLE 1:** Antibacterial and antifungal MIC values for AP aqueous extracts and positive controls.

	Gram-positive bacteria			Gram-negative bacteria				Fungus
Microbe name	*S. aureus*	MRSA	*E. faecium*	*E. coli*	*P. aeruginosa*	*K. pneumoniae*	*P. vulgaris*	*C. albicans*
Source	ATCC 25923	Clinically diagnosed	ATCC 700221	ATCC 25922	ATCC 27853	ATCC 13883	ATCC 700221	ATCC 90028
AP 100 μg/mL	3.125	6.25	6.25	6.25	3.125	6.25	6.25	R
Ampicillin	0.312	3.25	6.25	0.312	1.25	0.1	18	-
Ciprofloxacin	0.078	12.5	14	0.0156	0.0312	0.00125	0.15	-
Fluconazole	-	-	-	-	-	-	-	1.56

### Anticoagulant properties

The *in vitro* coagulation assay results showed that the aqueous extract of AP led to prolonged aPTT and TT test results in a dose-dependent manner, with the highest effect at 50 μg/mL. At the same time, a significant increase in PT test time was observed only at a high concentration of plant extract (50 μg/mL), as shown in Tables 2–4. These tests are used to evaluate the coagulation cascade. PT is used for the evaluation of the extrinsic and common pathways of the coagulation cascade. APTT is used to assess the intrinsic and common pathways, while TT is used to evaluate the conversion of fibrinogen to fibrin in the common pathway (Omar et al., 2017; Abdul Rehman et al., 2019), as described in the Methods section. Our results suggested the inhibition of clotting factors in the intrinsic and common pathways because both aPTT and TT tests were significantly prolonged. The inhibition increased in these tests as the concentration of plant extract increased. PT was slightly prolonged at high concentrations of plant extract as a result of an inhibition of the common pathway, mainly through the inhibition of the intrinsic and common pathways.

**TABLE 2:** Activated partial thromboplastin time (aPTT) results (in seconds) for AP aqueous extracts.

Sample	Participant 1 mean ± SD	Participant 2 mean ± SD	Participant 3 mean ± SD	Participant 4 mean ± SD	Participant 5 mean ± SD
Plasma only	25.3 ± 0.1	26.2 ± 0.1	30.45 ± 0.05	27.95 ± 0.05	29.15 ± 0.05
Plasma + PBS	25.65 ± 0.05	26.85 ± 0.05	31.2 ± 0.1	28.6 ± 0.1	29.75 ± 0.05
Plasma + 1% DMSO	25.6 ± 0.1	26.9 ± 0.1	31.35 ± 0.05	28.7 ± 0.1	29.8 ± 0.1
Aqueous extract					
Plasma +50 μg/mL	>200	>200	>200	>200	>200
Plasma + 25 μg/mL	185 ± 4	166 ± 3	179 ± 5	147 ± 6	163 ± 4
Plasma + 5 μg/mL	45.3 ± .6	46.6 ± 0.5	48.6 ± 0.2	45.9 ± 0.4	44.65 ± 0.15
Plasma + 1 μg/mL	31.6 ± 0.2	33.5 ± 0.3	37.7 ± 0.2	34.85 ± 0.25	32.1 ± 0.1

**TABLE 3:** Thrombin time (TT) results (in seconds) for AP aqueous extracts.

Sample	Participant 1 mean ± SD	Participant 2 mean ± SD	Participant 3 mean ± SD	Participant 4 mean ± SD	Participant 5 mean ± SD
Plasma only	10.25 ± 0.05	10.1 ± 0.1	12.35 ± 0.05	10.1 ± 0.1	11.3 ± 0.1
Plasma + PBS	10.45 ± 0.05	10.55 ± 0.05	12.6 ± 0.1	10.35 ± 0.05	11.65 ± 0.05
Plasma + 1% DMSO	10.4 ± 0.1	10.6 ± 0.1	12.7 ± 0.1	10.4 ± 0.1	11.7 ± 0.1
Aqueous extract					
Plasma +50 μg/mL	38.9 ± 0.2	41.7 ± 0.1	39.7 ± 0.2	35.1 ± 0.2	36.7 ± 0.1
Plasma + 25 μg/mL	19.3 ± 0.1	23.3 ± 0.1	22.4 ± 0.1	18.6 ± 0.2	20.4 ± 0.1
Plasma + 5 μg/mL	10.85 ± 0.05	11.25 ± 0.05	12.85 ± 0.05	10.4 ± 0.1	12.15 ± 0.05
Plasma + 1 μg/mL	10.45 ± 0.05	10.55 ± 0.05	14.75 ± 0.05	10.3 ± 0.1	11.65 ± 0.05

**TABLE 4:** Prothrombin time (PT) results (in seconds) for AP aqueous extracts.

Sample	Participant 1 mean ± SD	Participant 2 mean ± SD	Participant 3 mean ± SD	Participant 4 mean ± SD	Participant 5 mean ± SD
Plasma only	13.25 ± 0.05	13.1 ± 0.1	14.55 ± 0.05	12.25 ± 0.05	12.2 ± 0.1
Plasma + PBS	13.55 ± 0.05	13.35 ± 0.05	14.85 ± 0.05	12.65 ± 0.05	13.1 ± 0.1
Plasma +1% DMSO	13.65 ± 0.05	13.35 ± 0.05	14.85 ± 0.05	12.7 ± 0.1	13.15 ± .05
Aqueous extract					
Plasma + 50 μg/mL	19.5 ± 0.1	18.85 ± 0.05	18.95 ± 0.05	17.0 ± 0.1	18.35 ± 0.05
Plasma + 25 μg/mL	15.55 ± 0.05	15.4 ± 0.1	16.4 ± 0.1	14.6 ± 0.1	15.25 ± 0.05
Plasma + 5 μg/mL	13.65 ± 0.05	13.9 ± 0.1	15.15 ± 0.05	13.35 ± 0.05	13.85 ± 0.05
Plasma + 1 μg/mL	13.5 ± 0.1	13.4 ± 0.1	15.0 ± 0.1	12.8 ± 0.1	13.2 ± 0.1

### *Arum palaestinum* fractions inhibit the DNA cell cycle of Hep3B cells

Cell cycle parameters were investigated following treatment with aqueous, DMSO, and methanol fractions of AP flowers. The Hep3B cells were incubated with these fractions for 48 h. Doxorubicin (DOX), an anti-cancer drug known to inhibit cell proliferation and DNA arrest, was used as a control.

**FIGURE 1:**
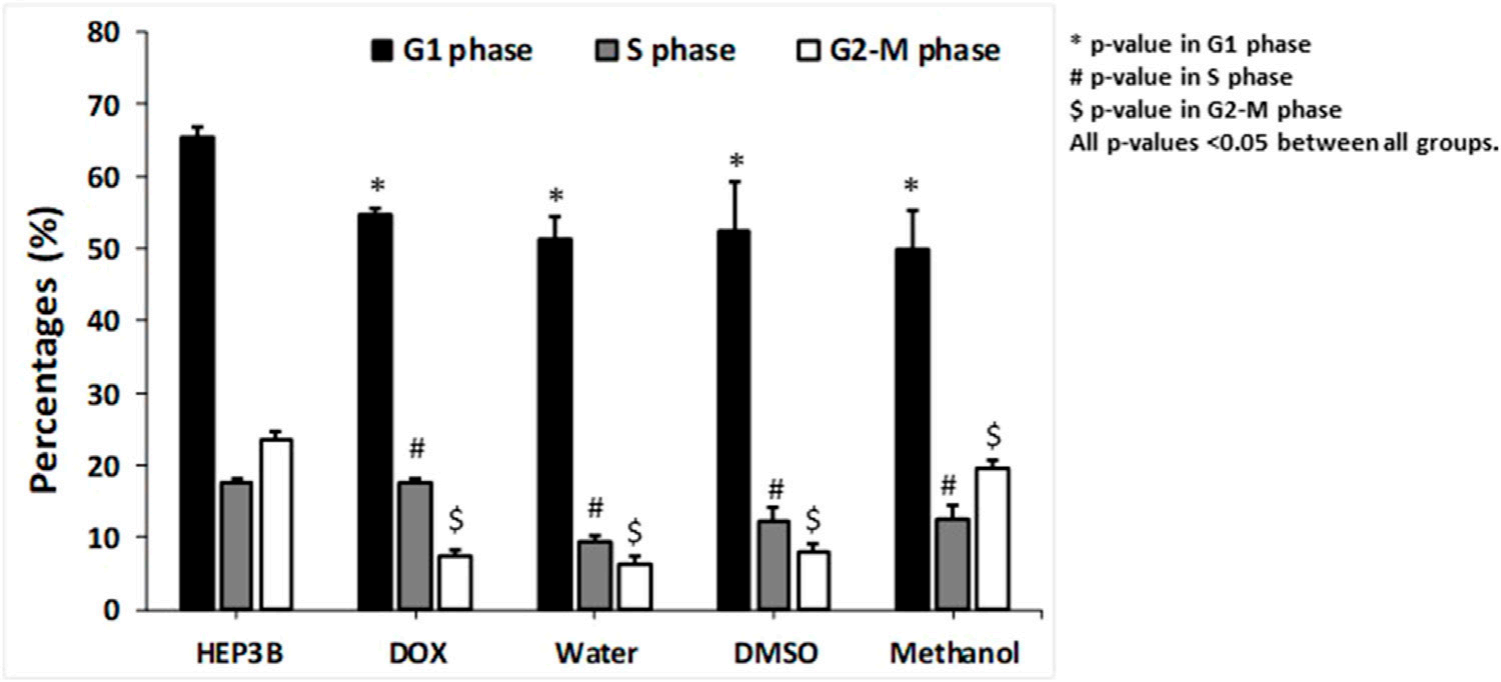
Proportions of cells for each cell cycle phase after exposure to the different fractions of the AP flower extracts. Cell cycle staining with PI, as described in Materials and Methods. The figure shows averages of three different readings of percentages of cells in the G1 phase, S phase, and G2-M phase, following treatments with different extracts of AP. *p* value < 0.05 between all groups.

As shown in Figure 1, DOX-treated cells showed a decrease in cells in the G1 phase, from 65.3% ± 7.8 in untreated cells to 54% ± 5.1% (*p* = 0.01). The cells treated with AP fractions also showed a shift in G1 populations to 51.3% ± 3.1%, 52% ± 2.2%, and 50% ± 2.4% in the aqueous, DMSO, and methanol fractions, respectively (*p* < 0.05 in all groups). Moreover, the cells treated with AP fractions also showed a shift in the S-phase population, which oversees DNA replication, from 17.7± in both Hep3B and DOX-treated cells, to 9.5% ± 2.6%, 12.1% ± 1.9%, and 12.6% ± 1.5% in the aqueous, DMSO, and methanol AP fractions, respectively (*p* < 0.05 in all groups). The same pattern of results was obtained in the G2-M-phase populations. The AP fractions inhibited the population of cells in the G2-M phase, during which mitosis commonly occurs, from 23.7% ± 1.5% in the untreated cells to 6.35% ± 1.9%, 8.1% ± 3.1%, and 19.7% ± 5% in the aqueous, DMSO, and methanol fractions, respectively (*p* < 0.05 in all groups). The G2-M phase in DOX-treated cells comprised 7.4% ± 1.8% of the population. Overall, the data indicated a delay in the cell cycle through decreased cell proliferation rates, following the effects of the AP fractions. The delay in the S phase favored the water fraction. The water and DMSO fractions maintained the cells in the G2-M phase, similar to DOX, while the AP extract in methanol accelerated cells in the G2-M phase. Therefore, these data suggest the anti-cancer properties of AP extracts.

### *Arum palaestinum* aqueous fractions reduce cell viability and shift cells to apoptosis

**FIGURE 2:**
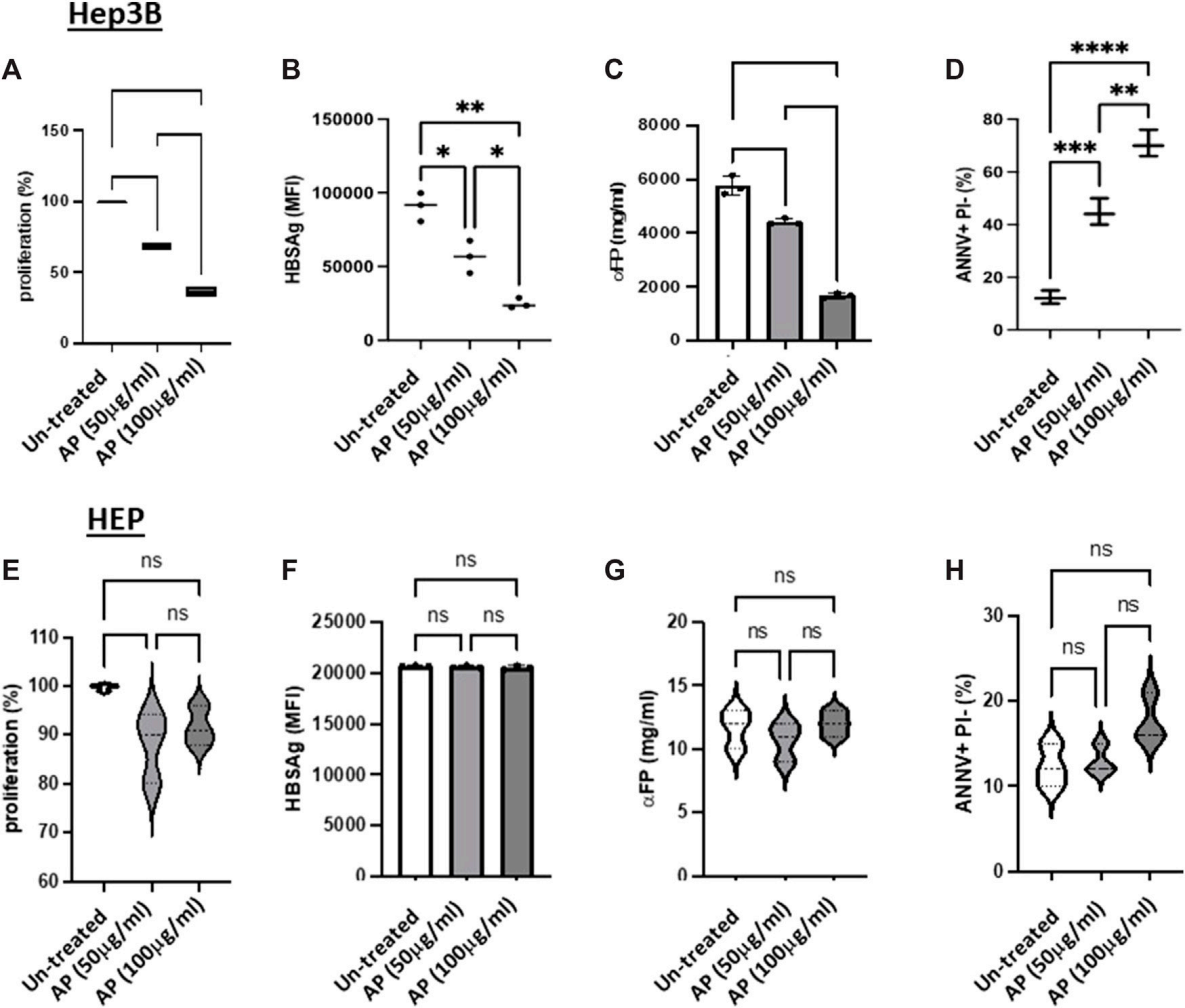
*Arum palaestinum* aqueous fractions alleviate cell viability and shift cells to apoptosis. Proliferative **(A)** and tumorigenicity **(B,C)** and apoptotic markers of HCC **(D),** following treatments with AP. The cells were treated with 50 and 100 μg/mL of AP aqueous extracts. **(E–H)** Normal primary hepatocytes were used as controls. The averages ±SD are shown. For statistically significant differences, paired and unpaired Student’s t-test and analysis of variance were used. *p* value < 0.05 between all groups.

**FIGURE 3:**
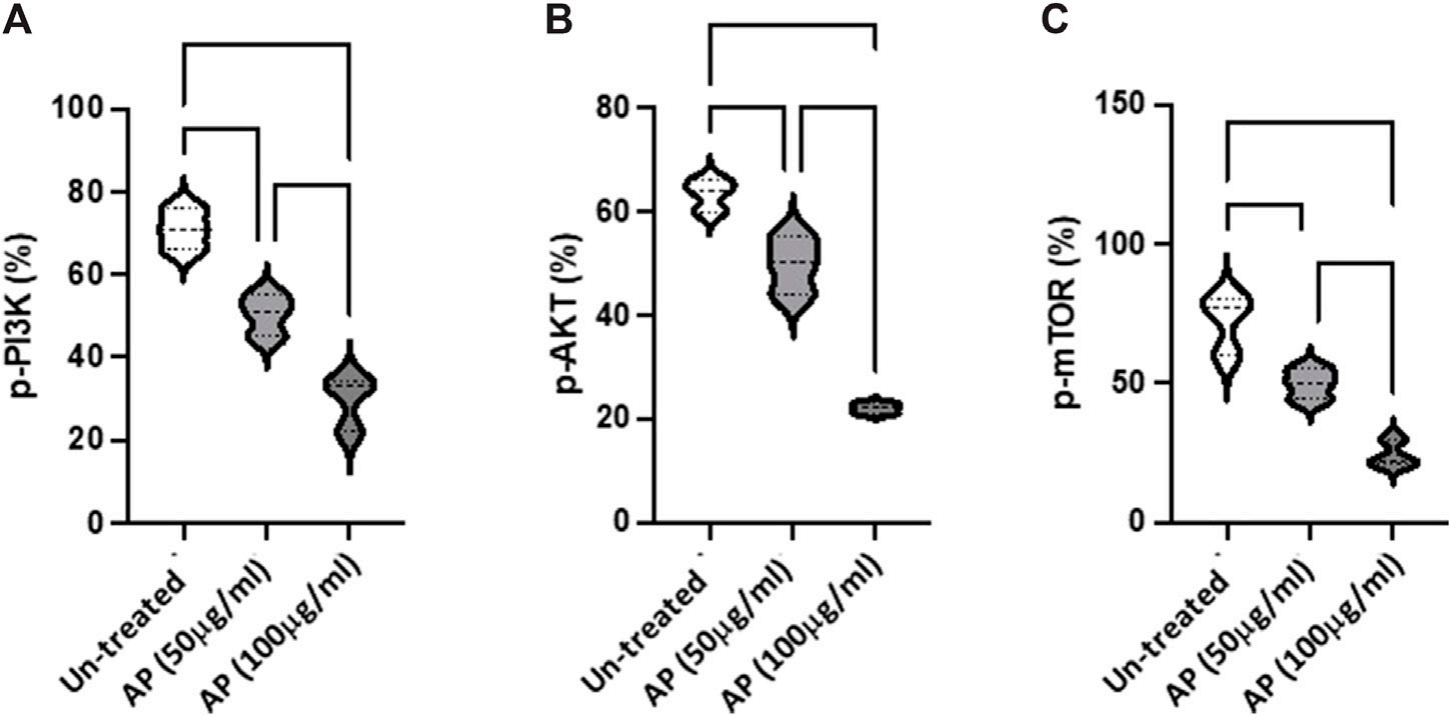
*Arum palaestinum* inhibits PI3K/AKT/mTOR signaling. Flow cytometry analysis of the **(A)** p-PI3K (Tyr 458), **(B)**, p-AKT (S473), and **(C)** p-mTOR (S2448) signaling pathways in HCC cells with and without treatment with 50 or 100 μg/mL of AP. *p* value < 0.05 between all groups.

We next assessed the effects of the AP extracts on Hep3B cell proliferation, as well as their anti-cancer activities (through the measurement of αFP secretions and cell HBsAg expression). We chose the aqueous fractions with concentrations of 50 and 100 μg/mL for performing proliferations, cancerogenesis potentials and apoptosis experiments. The cells were seeded in 6-well plates at a density of 1 × 10^6^ cells/well. The effects of AP on cell viability and proliferation were determined by CSFE assay. Figure 2 demonstrates a significant and dose-dependent inhibition in Hep3B cell proliferation and cancer activity, following AP treatment, compared with untreated cells. The AP treatments decreased proliferation by up to 50% and 40% for AP concentrations of 50 and 100 μg/mL, respectively. It has been well documented that Hep3B cells have 1–2 copies of hepatitis B viral DNA integrated into the host genome. Hep3B cells also continue to synthesize and secrete HBsAg’ and αFP into culture media. The cells treated showed decreasing HBsAg levels by up to 50.7% and 80%, following treatment with 50 and 100 μg/mL of AP, respectively (Figure Figure2B). Figure 2C shows that the average αFP levels declined to 4,425.8 ± 64.8 ng/mL and 1,168.3 ± 88.4 ng/mL, following AP treatment, respectively, compared to the average of 5,762.2 ± 198.1 ng/mL in the untreated cells. We then assessed the effects of AP in disturbing the DNA content and consequently provoking programmed cell death (apoptosis). As cells going through apoptosis show movement of phosphatidylserine (PS) phospholipid from the inner face of the plasma membrane to the cell surface, apoptotic cells can be identified by the presence of PS on the cell surface. As described in the Methods section, PS was stained with a fluorescent conjugate of Annexin-V, which is a protein with a high affinity for PS. After staining, flow cytometry analysis was performed. The cells were also stained with propidium iodide (PI), which can enter the cell only when the plasma membrane is impaired. Early apoptosis was defined as PS positivity but PI negativity. Figure 2D demonstrates that the untreated cells (Hep3B cells alone) maintained a baseline apoptotic cell population of 10% ± 5.3%, while cells treated with AP at concentrations of 50 and 100 μM showed elevation in the apoptotic population to 40.3% ± 2.5% and 70.3% ± 1.5%, respectively (*p* < 0.05). Generally, these data suggest that AP flowers shifted the cells from necrosis to apoptosis by delaying the G2-M phase of the Hep3B cell cycle and that, together with the results in Figure 1, suggest anticancer potential following incubation with AP extracts. Our results displayed no significant changes in the detected parameters when using the normal liver primary hepatocytes (Figures 2E–H). Supplementary Figure S1A displays a representative confocal microscopy image of normal primary hepatocytes obtained from the biopsies of the human liver in patients with hepatocellular carcinoma. DAPI was used for nucleus staining (blue) and anti-albumin (yellow). In Supplementary Figure S1B, semi-confluent primary hepatocytes were observed in the culture medium, following 7 days of isolation, as described in the Methods section (Figure 3).

### *Arum palaestinum* inhibits the PI3K/AKT/mTOR signaling pathway

To further evaluate the molecular changes resulting from the effects of AP in HCC, we assessed the PI3K/AKT/mTOR pathway as a major intracellular signal transduction pathway involved in regulating the cell cycle, cell proliferation, apoptosis, metabolism, and angiogenesis The flow cytometry results indicated the elevated phosphorylation of PI3K, AKT, and mTOR in Hep3B were inhibited in a dose-dependent manner, following AP treatment (50 μg/mL, *p* < 0.05; 100 μg/mL, *p* < 0.01; Figures 3A–C). The obtained dephosphorylated pathway provided further evidence on the delay of cell proliferation and tumorigenicity (however, without affecting cell viability (data not shown).

**FIGURE 4:**
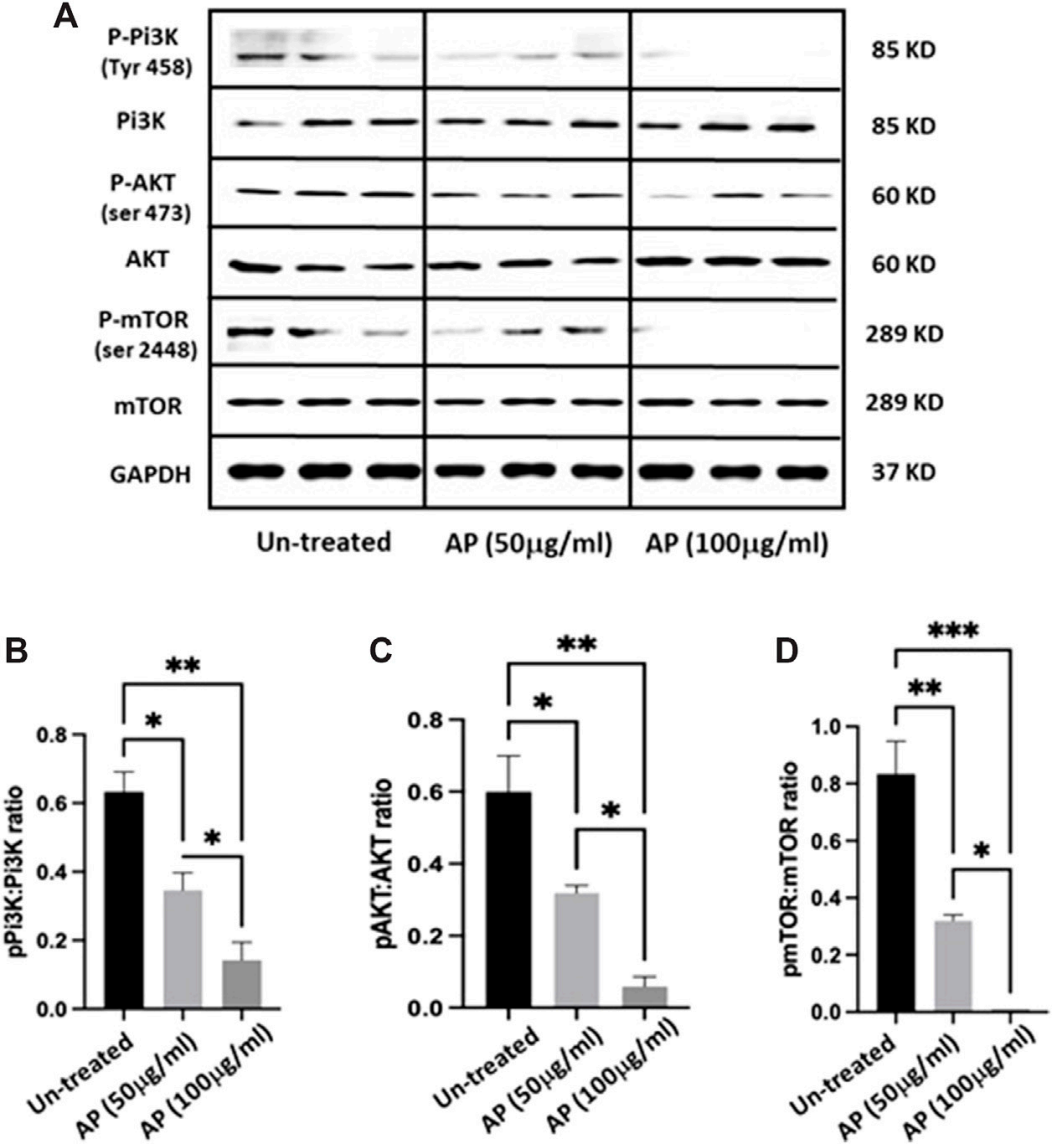
*Arum palaestinum* effects on the PI3K/AKT/mTOR phosphorylation signaling pathway. Representative Western blot analysis of three repeated samples of **(A)** p-PI3K (Tyr 458), p-AKT (S473), and p-mTOR (S2448) assessed in Hep3B cells with and without treatment with 50 or 100 μg/mL of AP. Quantitation of **(B)** p-PI3K, **(C)** p-AKT, and **(D)** p-mTOR through phosphorylated proteins to measure the protein ratios using SOLOFusion analysis. *p* value < 0.05 between all groups.

Figure 4A shows densitometry bands of a Western blot of representative proteins of p-PI3K, p-AKT, p-mTOR, and the GAPDH housekeeping protein. The average quantitation values of the measured proteins are presented in Figures 4B–D. The data showed decreased phosphorylation of PI3K, AKT, and mTOR proteins, following exposure to the AP extracts (*p* < 0.05). This mechanistic approach may suggest a new metabolic impacts of AP, which could be used as a future approach for therapy related to metabolic profile.

## Discussion

Herbal plants have been traditionally used for multiple medical purposes. Many herbal remedies have potential and beneficial effects on health and disease as they can control, prevent, and treat various biological disorders, from the simplest to the most complicated. *Arum palaestinum* (AP), a Mediterranean-region plant, has been conventionally applied as a medical remedy for the treatment of diverse illnesses such as constipation, bacterial infections, blood disorders, and cancer. Despite these uses, there is a lack of knowledge and studies on the biological roles of the AP plant; therefore, a deeper understanding of its vital actions is required (Ali-shtayah et al., 2008).

Herein, we first identified the antimicrobial activity of AP aqueous extracts against selected strains of *S. aureus*, MRSA, and *E. faecium* as Gram-positive bacteria; *K. pneumoniae*, *P. vulgaris*, *E. coli*, and *P. aeruginosa* as Gram-negative bacteria; and *Candida albicans* as a fungal strain. Our results showed the relevant antimicrobial activity of AP extracts against all bacterial strains; however, the extracts showed a significant decrease in MIC values by approximately three-fold in the *P. vulgaris* strain compared to the ampicillin control groups, and by approximately two-fold in the MRSA and *E. faecium* strains compared to the ciprofloxacin control groups. In contrast, there was no notable effect on the *C. albicans* fungal strain. The effective antibacterial results obtained from the AP extracts show that AP can contribute beneficially to alleviating an expanding global health issue related to multi-drug-resistant bacteria. Although there is a huge progression in the synthesis of antibacterial drugs, the risk of multi-drug resistance is also increasing, leading to harmful and lethal effects. Combination therapies of AP extract with other antibacterial medications can, therefore, reduce resistance and mortality rates and improve patient quality of life (Basavegowda and Baek, 2022).

It is widely known that the coagulation process is a dynamic cascade of events involving multiple clotting factors and pathways that promote stable clot formation. An imbalance in the homeostasis of blood fluidity will lead to blood disorders; among these, hypercoagulability is a serious health issue that promotes thrombosis and strokes, leading to death (Palta et al., 2014).

In our study, we observed the anti-coagulant activity of AP extract on extrinsic, intrinsic, and common coagulation pathways through PT, APTT, and TT testing of blood samples. The PT test is known to target the extrinsic and common pathways, while the APTT and TT tests target the intrinsic and common pathways. The results showed that the AP extract produced significantly prolonged times in the APTT and TT tests in a dose-dependent manner compared to the PT test, which conferred valuable activity only at the highest doses of AP extract.

The anti-coagulability effects of the AP extract through inhibition of either the intrinsic and common pathways or both could be a potential approach for inhibiting protein C pathway, as this pathway is used to inhibit factor Va (in the common pathway) and factor VIIIa (in the intrinsic pathway). This plant may contain active ingredients that activate protein C to its activated form (APC), while APC inhibits factors Va and VIIa to cause the observed prolonged results in the intrinsic and common pathways (Ayodele et al., 2019). These findings could suggest a role of AP as an anti-thrombin therapy (Abdul Rehman et al., 2019). Furthermore, in this study, we observed the anti-cancer activity of AP extracts. It is well-known that most anticancer agents directly or indirectly target cell cycle and proliferation rates. Thereby, we tested the AP extracts in hepatocellular carcinoma and normal primary liver cell lines to analyze any delay in the cell cycle or changes in cell proliferation. Our results did not show any effect on normal primary liver cell lines; however, we observed a significant decrease in the hepatic cancer cell cycle. Cells treated with AP extract showed diminished populations in the G1 and S phases, with a larger effect in the G2-M phases compared to the negative and doxycycline control groups. Consequently, we tested if the delay in cell cycle phases would promote programmed cell death, the so-called apoptosis process. The results of cell viability assays performed after treating the cell lines with AP extracts showed a large increase in the number of apoptotic cells compared to the control groups. These results support the conclusion that the potential delay in the G2-M phase specifically shifts cells from necrosis to apoptosis upon treatment with AP extracts in a dose-dependent manner.

Moreover, we assessed the anti-tumorigenic effect of AP extract by measuring the expression levels of αFP and HBsAg tumor markers in hepatocellular carcinoma. αFP is naturally expressed in liver cells during cell regeneration in fetal liver development or inflammatory disorders such as hepatitis and cirrhosis. However, as elevated levels of αFP are associated with hepatocarcinoma in adults or hepatoblastoma in infants, it is a useful tumor marker protein (Galle et al., 2019). Similarly, HBsAg is expressed following infection with hepatitis B virus, and extreme levels of this protein are also associated with HCC (Xie, 2017). Significant dose-dependent decreases in expression levels of both tumor markers were observed upon treatment with AP extract. These results were consistent with our previous findings illustrating the cytotoxic activity of AP extract to promote cancer cell death.

Mechanistically, we assessed the molecular activity of AP extracts in promoting cell death in a hepatocellular carcinoma cell line. Given the fact that the PI3K/AKT/mTOR pathway is a major signaling pathway that is abnormally activated in various cancer types, including liver cancer, wherein it promotes cell proliferation, growth, and angiogenesis that are vital for cancer metastasis and development. The PI3K/AKT/mTOR pathway is naturally regulated by phosphorylation/dephosphorylation cascades from receptor tyrosine kinases present on the cell surface far down to the gene expression within the nucleus (Wang et al., 2022). Our results showed significant dose-dependent inhibitions in the phosphorylation levels of the PI3K, ATK, and mTOR proteins in the cancer cell lines upon treatment with AP extract. These results further promote the conclusion of the potential cytotoxic and anti-cancer activity of the AP extract *in vitro*, which is most likely mediated through the PI3K/AKT/mTOR pathway. However, further studies are warranted to better understand the more precise downstream cell signaling regulated by the AP extract in cancer cells and in a preclinical *in vivo* liver cancer model to promote the use of AP extract in future therapeutic studies.

## Conclusion

To the best of our knowledge, the present study is the first report on the cytotoxic activities of AP together with its related associations. These findings inform the future development of naturally occurring antiplatelet and antibacterial medications, although some synergistic or antagonistic effects require clarification. Moreover, the targeting of the molecular pathways related to cancer activity also needs further study.

## Data Availability

The original contributions presented in the study are included in the article/Supplementary Materials, further inquiries can be directed to the corresponding authors.

## Supplementary material

Supplementary Figure S1
**(A)** Representative confocal microscopy image of normal primary hepatocytes obtained from the biopsies of human livers from patients with hepatocellular carcinoma. DAPI, nucleus staining (blue); anti-albumin (yellow). **(B)** Semi-confluent primary hepatocytes in culture medium, following 7 days of isolation, as described in the Methods section.
