# Supplementary figures and images for: Arum palaestinum delays hepatocellular carcinoma proliferation through the PI3K-AKT-mTOR signaling pathway and exhibits anticoagulant effects with antimicrobial properties
Source: Front Pharmacol. 2023 Jun 1;14:1180262. doi: 10.3389/fphar.2023.1180262 (PMC10270306; doi:10.3389/fphar.2023.1180262)

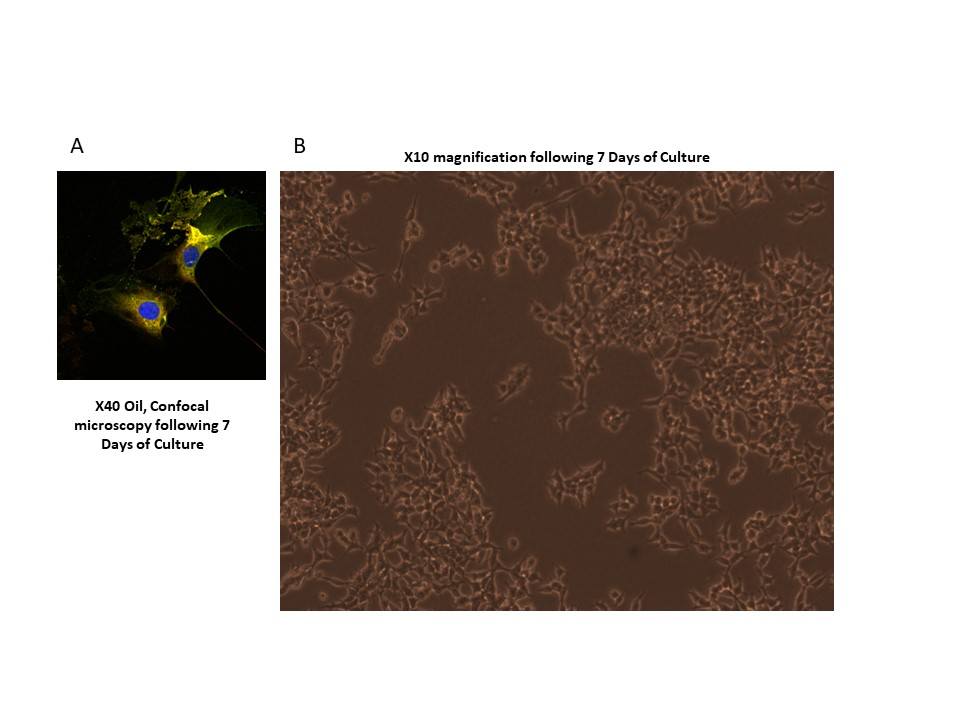

Supplement: Supplementary file 1 [file Image1.jpeg]
